# *In Silico*, *In Vitro*, and *In Vivo* Wound Healing Activity of *Astragalus microcephalus* Willd.

**DOI:** 10.1155/2022/2156629

**Published:** 2022-10-04

**Authors:** Fatemeh Akbari, Mohammad Azadbakht, Ali Bagheri, Lale Vahedi

**Affiliations:** ^1^Department of Pharmacognosy, Faculty of Pharmacy, Mazandaran University of Medical Sciences, Sari, Iran; ^2^Department of Plant and Animal Biology, Faculty of Biological Science and Technology, University of Isfahan, Isfahan, Iran; ^3^Department of Pathology, Faculty of Medicine, Mazandaran University of Medical Sciences, Sari, Iran

## Abstract

**Methods:**

The methanolic root extract was prepared by maceration, and flavonoids were evaluated by LC/MS. *In silico* examination was performed based on the LC/MS results, and the binding affinity of these compounds to estrogen receptors (ERs) *α* and *β* was evaluated. Wound healing evaluation in both *in vitro* (NHDF cell line, by 500 *μ*g/ml concentration of the extract, 24 h) and *in vivo* (Wistar rat, topical daily treated with 1.5% of the extract ointment, 21 days) conditions in comparison to control groups was conducted. Rats' control groups included silver sulfadiazine, Vaseline, and the nontreated groups.

**Results:**

Eleven flavonoids were detected using LC/MS. The *in silico* study showed that formononetin, kaempferol-based structures, quercetin-3-O-neohesperidoside, and calycosin-7-O-beta-D-glucoside had a high affinity (<−6.3) to ERs *α* and *β*. Wound closing measurement showed significant improvement in the group treated with the extract in both *in vitro* and *in vivo* assays compared to the control groups. Histopathological results confirmed these findings; inflammation factors decreased, and fibroblast proliferation, fibrosis, and epithelization increased, especially in the extract group.

**Conclusion:**

This study shows that *Astragalus microcephalus* has wound healing activity *in vitro* and *in vivo* with low toxicity due to the presence of flavonoids, especially isoflavonoids, which show a high affinity to bind to ERs *α* and *β* in the skin tissue.

## 1. Introduction

Skin is an important barrier that protects the body from environmental hazards such as infections, radiation, and tissue trauma. A wound is one of the high-prevalence complications. In 2018, it was reported that 8.2 million people had wounds with or without infections and a general population of 2.21 per 1,000 suffer from chronic wounds [1, 2].

Underlying diseases such as diabetes or infections make wounds a life-threatening problem [3]. Wound care has been traced back to the earliest civilizations, and the ancient Greeks were among the first to emphasize the importance of wound healing. Adequate wound care prevents infection and other complications and helps accelerate the healing process by reducing scarring [4].

Wound healing includes an arranged progression of events that establish the integrity of damaged tissue. The healing process consists of four biological processes: hemostasis, inflammation, proliferation, and remodeling [5]. Both dermal and immunity cells are involved in the process. Immunity cells include neutrophils, macrophages, and lymphocytes; dermal cells include keratinocytes, fibroblasts, and endothelial cells [6].

Local and systematic factors influence the wound healing process. Local factors include venous sufficiency, foreign body, infections, and oxygenation. Systematic factors can be underlying disease, medications, aging, sex hormones, and any condition that suppresses the immune response. Sex hormones, including estrogens (estrone and 17*β*-estradiol) and androgens and related hormones (testosterone and 5*α*-dihydrotestosterone dehydroepiandrosterone), influence the wound healing process [7]. The difference in gene expression between the wounds of aged and young individuals is regulated almost exclusively by estrogen [8], whereas androgens negatively affect wound healing [9].

Estrogens play a role in wound healing by regulating various genes involved in regeneration, matrix production, protease inhibition, epidermal function, and genes primarily associated with inflammation [10].

The treatment of a wound consists of supportive therapies such as cleansing the wound area, removing dead skin surgically, closing large wounds with stitches or staples, bandaging the wound, relieving pain, and treating signs of infection with medications. Many studies have evaluated various chemical and natural compounds to find proper and effective solutions for wound complications. Despite all the efforts to find a solution, there remains a need to find an effective treatment to accelerate the wound healing process, heal the scars, and return the tissue function.

*Astragalus* is a large genus with more than 3,000 species worldwide, especially in the Northern Hemisphere. More than 800 species of this genus (about 10% of the Iran's plant species) grow in Iran [11, 12]. These species belong to the Leguminosae or Fabaceae family and constitute the most abundant genus among the plant species. These species include herbs and shrubs, which contain annual and perennial forms. The leaves of the species are compound, and the flowers are butterfly-like [13]. *Astragalus* has a long history of use in traditional medicine in most countries where it has been able to grow. In Iranian traditional medicine, this plant is referred to as “Gavan,” “Ghatad,” “Taraghafitha,” “Targhaqia,” “Targhaghia,” and “Targhaghant” [12, 14]. The most important pharmacological contents of the roots of the Fabaceae family are polysaccharides, saponin, and flavonoids, specially isoflavonoids.

Isoflavonoids are flavonoid classes with the basic 3-phenylchromen-4-one structure; they are found in two forms: glycoside and nonglycoside (aglycone). These compounds have estrogen-like structures. Isoflavonoids can bind to the human estrogen receptors (ERs) and exhibit partial agonist effects [15, 16].

In the current study, *Astragalus microcephalus *Willd. (a. mic) was phytochemically evaluated. The *in vitro* and *in vivo* effect on wound healing was conducted by focusing on the activity of its probable isoflavonoids and its phytoestrogen properties. Furthermore, the bond affinity of identified isoflavonoids to ERs *α* and *β* was examined by molecular docking.

## 2. Materials and Methods

### 2.1. Plant Material

*Astragalus microcephalus* roots were collected from the growing areas in the Zanjan province, the northern slopes of the Qeydar Mountains (latitude 36° 07′N; longitude 48° 34′E) according to the relevant literature [17, 18]. Herbarium samples were prepared and kept at the Herbarium of the University of Isfahan (herbarium code: 23020), Zanjan province.

### 2.2. Extraction

*Astragalus microcephalus*'s root (2.5 kg) was extracted by maceration with absolute methanol at room temperature for 3 days, repeated three times. The extract was dried under vacuum by a rotary evaporator. The extract was kept at −15°C.

### 2.3. Preparation of the Herbal Ointment

For preparing 1.5% ointment of *Astragalus microcephalus* extract, 7.5 g of extract was dissolved in a minimum amount of water and added to Eucerin (lanolin alcohol, levigating agent) and then gradually to ointment base, Vaseline, up to 500 g.

### 2.4. Total Flavonoids Content Determination

The spectrophotometry method was used to evaluate flavonoid content with the aluminum chloride colorimetric method [19].

### 2.5. HPLC Analysis of Genistein

Isolation of isoflavones was performed by the method of Lozovaya et al. [20]. A C18 column with dimensions of 53 × 7 mm was used. The gradient solvent system consisted of water-acetic acid (1-liter deionized water with 1.2 ml acetic acid) and acetonitrile. The injection volume was 20 microliters, and the acetonitrile solvent was added from 0% to 12% for 6 min and then raised from 23% to 100% for 15 min. The solvent flow was performed at 2.5 ml/min at 280 nm using a UV detector. Standard genistein (external standard; Merk, Germany) and standard mixed with the extract (internal standard) were separately injected into the system to compare and measure the amount of this compound in the plant.

### 2.6. LC/MS Evaluation

*Astragalus microcephalus* was dissolved with the minimum amount of water and then filtrated with a 0.45 *μ*m pore size filter. 10 *μ*L of the extract was injected into Agilent 6100 Quadrupole LC/MS system to detect flavonoids, which were checked with library standard chromatogram in the system using the diode array detector SL (micro flow cell: 2 *μ*L, 3 mm path length).

LC/MS was conducted according to the Lin method [21]. For this purpose, an Agilent series 6100 LC/MS system (Agilent Technologies, Santa Clara, USA) with a photodiode array detector was set at 260 nm. The UV spectra were examined at 200–500 nm to obtain the maximum absorbance wavelength. A 150 × 3.0 mm, 3.5 *μ*m Waters XTerra MS with a sentry guard column (Symmetry C18, 5 *μ*m, 20 × 3.9 mm) was used. The mobile phase contained (1) water with 0.25% (v/v) acetic acid and (2) acetonitrile with 0.25% (v/v) acetic acid using a linear gradient of 17%–42% (v/v) (2) for 38 min. The temperature and flow rates were set to 45°C and 0.2 ml/min, respectively.

The LC system was joined to the mass spectrometer directly without stream splitting and using an electrospray interface Model HP 59987A. The ESI-MS spectra were obtained from the positive ion mode. The nebulizer pressure (N2) was 5.5 × 105 Pa, and the temperature of the dying gas (N2) was set to 350°C, with a gas flow rate of 40 ml/min.

### 2.7. Molecular Docking

The crystal structures of ERs *α* and *β* with Protein Data Bank (PDB) ID of 1R5K and 1X7B, respectively, were retrieved from the protein databank (https://www.rcsb.org). The crystal structures were prepared by removing the existing ligand while missing hydrogen atoms were added using BIOVIA Discovery Studio 2020. Thereafter, nonpolar hydrogens merged, while polar hydrogens were added to the enzyme. The enzyme was saved into dockable Protein Data Bank, Partial Charge, and Atom Type (PDBQT) format in preparation for molecular docking.

Furthermore, SDF structures of formononetin (CID 5280378), kaempferol-3-O-glucoside (CID 5282102), kaempferol-3-O-sophoroside (CID 5282155), kaempferol-3-O-neohesperidoside (CID 5318761), quercetin-3-O-neohesperidoside (CID 5748416), calycosin-7-O-beta-D-glucoside (CID 71571502), estradiol (CID 5757) as the standard agonist and finasteride (CID 57363) as the standard antagonist were retrieved from the PubChem database (https://www.pubchem.ncbi.nlm.nih.gov). The compounds were converted to PDB format using BIOVIA Discovery Studio 2020. These ligand molecules were further converted to the dockable PDBQT format using Autodock tools.

The molecular docking procedure was performed by docking formononetin, kaempferol-3-O-glucoside, kaempferol-3-O-sophoroside, kaempferol-3-O-neohesperidoside, quercetin-3-O-neohesperidoside, and calycosin-7-O-beta-D-glucoside for ER alpha and beta, using the *in silico* docking approach.

For validation and realistic results, this study used estradiol and finasteride as the standard ligands for comparison. Every single analysis was repeated three-time and the mean and standard deviation were calculated.

For this purpose, PyRx.lnk software was used, and binding affinities were determined.

The enzymes and ligands were dragged into their respective columns in their PDBQT form, and the software was launched.

The preferable computational method for determining the binding mode is using docking followed by free energy calculations. This predicts compounds' binding to receptors [22].

A cluster analysis was ultimately performed based on root mean square deviation (RMSD) values with reference to the starting geometry, and the lowest energy conformation of the more populated cluster was considered the most trustable solution. The binding affinities of the ligands and enzymes were recorded. Molecular interactions between the compounds and ERs *α* and *β* were viewed with BIOVIA Discovery Studio 2020 software. The binding profiles were determined using receptor cavities identified using Discovery Studio Visualizer.

### 2.8. *In Vitro* Drug Release Study

Two rats were anesthetized, and the abdominal skin hair was removed. The abdominal skin was separated and held on normal saline for 12 h and then applied to the dialysis tube. The concentration of 1.5% was selected.

The *Astragalus* ointment was applied on the surface of the dialysis tube, and the undersurface was kept in contact with deionized water, placed hermetically, sealed, and soaked in a tube containing 35 ml of deionized water. Continuous shaking was applied with a heater stirrer and a magnet in the inner part. The temperature was set at 37 ± 0.5°C at 2, 4, 6, 8, 10, 12, and 24 h passing the start of the procedure. 5 ml of the sample from the solution was replaced with 5 ml of deionized water in the dialysis tube to keep their levels even. The sample was evaluated with a spectrophotometer at 415 nm based on quercetin measurement [23].

### 2.9. MTT Cell Viability Assay

Normal human dermal fibroblast (NHDF) cell was purchased from Royan Institute for Stem Cell Biology and Technology, and the cells were seeded in a 96-well microplate (1 × 104 per well) in RPMI-1640 (Biosera, China) culture medium supplemented with 10% fetal bovine serum (FBS, Biosera, China), 0.01 M of HEPES (4-[2-hydroxyethyl]-1-piperazineethanesulfonic acid) (Biosera, China), 1% mixture of antibiotic/antimycotic (Gibco, USA), 0.001 M of sodium pyruvate (Gibco, USA), and 0.02 M of L-glutamine (Biosera, China), then incubating in an incubator with a humidified atmosphere with 5% CO_2_ for 24 h. After 24 h, the medium was replaced with a medium supplemented with extracts with different concentrations of 125, 500, 1,000, and 2,000 *μ*g/ml.

MTT assay was performed after 24 and 48 h. Therefore, 10 *μ*l of 3-[4,dimethylthiazole-2-yl]-2,5-diphenyl tetrazolium bromide (MTT reagent) was added to the microplate and incubated for 4 h, and 90 *μ*L of formalin buffer was added to each well. Furthermore, 100 *μ*L (10%) SDS (Sodium dodecyl sulfate, Gibco, USA) in 0.01 M HCl as solubilization solution was added to each well and remained in the incubator at 37°C. After solubilization of purple formazan product overnight, microplates absorbance was measured at 570 nm with ELIZA reader (*A*1) [24].

*A*1: viability percentage of cells = (absorbance of extract treated cultures − absorbance of background control)/(absorbance of control cultures − absorbance of background control) × 100.

### 2.10. Cell Culture Condition and Wound Scratch Assay

NHDF cell line was maintained in RPMI-1640 culture medium with supplemented material including 10% FBS, 1% mixture of antibiotic/antimycotic, 0.01 M of HEPES, 0.02 M of L-glutamine, and 0.001 M sodium pyruvate. Subsequently, it was incubated in an incubator (37°C, humidified atmosphere with 5% CO2) [25].

The *in vitro* wound healing activity of the extract was evaluated by wound scratch assay [26] by measuring the size of the area scratched in the extract-treated group compared to the nontreated group over 48 h. NHDF cells were seeded in 6 wells of 12-well plates (4 × 104 cells/well) and cultured with the above condition to obtain monolayer cells in each plate. Then, the medium was removed, and central scraping was performed using the tip of the p200 micropipette. The wells were washed with PBS to remove the nonadherent cells and the cell residue after scratching. The specific concentration of the *Astragalus microcephalus* extract was selected after MTT assay (500 ppm extract powder), mixed and dissolved in RPMI-1640 medium, and added to the wells. As the control group, three wells were treated with supplemented RPMI-1640 culture medium. The plates were incubated at 37°C (5% CO2), and in the periods of 0, 2, 8, and 24 h, the scratches were evaluated under the microscope; the progress of wound healing was compared to the control group (nontreated group). The wound status was calculated manually by IC Measure software version 2.0.0.286, the Imaging Source, Germany software [26].

### 2.11. Experimental Wounding and Experimental Design

Healthy male rats weighing 200–220 g were taken from the Institute for Laboratory Animal Research of the Mazandaran University of Medical Science. The animals had free access to food and water and were kept under standard conditions (12 h light-dark cycle at room temperature). Animal rights were respected according to the principles of the Association for the Protection of Animal Rights [23].

The animals were anesthetized with a ketamine–xylazine mixture with a dose of 0.1 ml/100 g per body weight *via* intraperitoneal injection. The dorsal hairs of animals were cut, and a 2.5 × 2.5 cm circular shape area was measured. Then, the full-thickness wound was made with excision on the dorsal surfaces of all animals.

Forty wounded rats were divided into four groups (*n* = 10, each animal was kept in a separate cage). For 21 days, the groups were daily treated as follows: group one was topically treated with a 1.5% extract of *Astragalus microcephalus* ointment (extract group), group two was topically treated with silver sulfadiazine (sliver group) as the standard medicine, group three was topically treated with ointment base (Vaseline group), and group four did not receive any treatment as the normal control (free group) for 21 days.

The 1.5% extract of *Astragalus microcephalus* ointment was selected for the treatment group. This selection was based on published articles on this genus and the effective amount of extract used in the animal experimental procedure of the previous articles [27, 28].

### 2.12. Wound Size Analysis

Wound dimensions were measured on days 1, 4, 7, 11, 14, and 21. For this purpose, the wound sizes were drawn on transparent paper and transferred to the graph paper. The ratio of wound healing was calculated according to the following formula:

*A*2: the ratio of wound healing = (area of original wound − area of remaining wound)/area of original wound × 100.

### 2.13. Histopathological Evaluation

On days 4, 7, 14, and 21, three rats of each group were euthanized by chloroform inhalation, and the tissues were collected. Furthermore, the normal healthy skin of rats was removed for further comparison. All samples were kept in separate containers filled with 10% formalin and stained with hematoxylin and eosin and Masson's trichrome stain. The samples were sent to the histopathological laboratory to examine the factors including congestion, edema, necrosis, fibroblast proliferation, collagen formation, angiogenesis, and epithelialization.

### 2.14. Statistical Analysis

Statistical analyses were performed using SPSS 26 software. A significance level of 0.05 (*P* ≤ 0.05) was considered in all cases, and the results were represented by mean ± SEM. The variable's normality was assessed using the Shapiro–Wilk test. The one-way ANOVA and Tukey *post hoc* tests were also used. The nonparametric Kruskal–Wallis test analysis was performed when the data were not normally distributed.

## 3. Results

### 3.1. Total Flavonoids Determination

By the standard curve equation of quercetin (*y* = 0.0067*x* + 0.2365, *R*^2^ = 0.9712), the amount of flavonoid in the extract was measured, and considering the extraction efficiency (9%), it was calculated as 0.0921 g per 1 g of the root of *Astragalus microcephalus.*

### 3.2. HPLC Analysis of Genistein in the Extract

**Figure 1:**
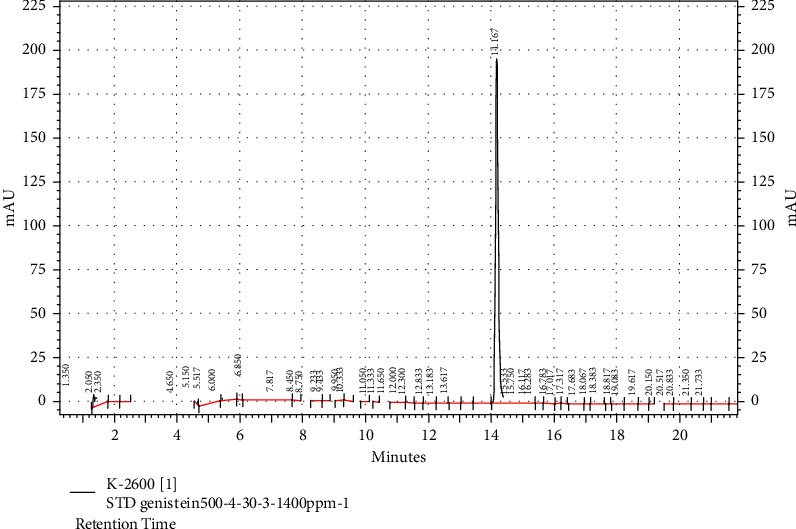
HPLC analysis of 500 ppm standard genistein. The standard peak of genistein is seen in retention time 14.167.

**Figure 2:**
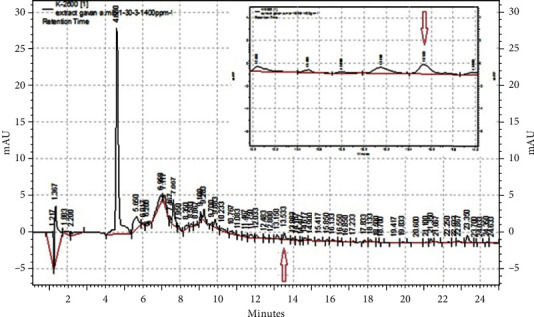
HPLC diagram of the extract with a concentration of 1,000 ppm. The red arrow indicates the location of the genistein peak in the extract.

**Figure 3:**
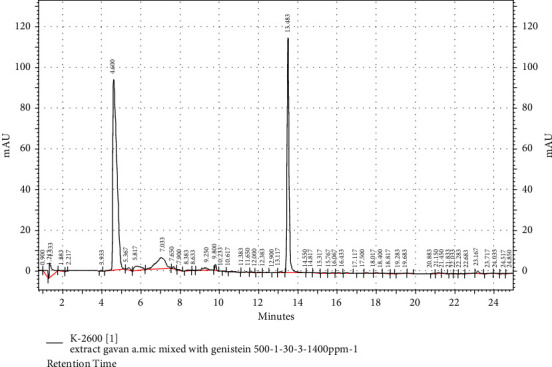
The HPLC diagram of the standard genistein mixed with the extract. Retention time 13.483 corresponds to the peak of genistein.

The diagrams of standard genistein, the extract, and the combination of the extract with the standard are presented in Figures 1–3, respectively. Genistein appeared with a retention time of 14.167 and 13.483 min in the standard diagrams of genistein and the combination of the extract with standard, respectively (Figures 1 and 3). It can be observed with a retention time of 13.353 min in the extract group, which is shown with a red arrow in Figure 2. Furthermore, with the standard curve of the standard genistein (*Y* = 2751.9*X* + 7414, *R*2 = 0.9905), the amount of area below the extract curve at the time of genistein peak appearance is 1.2449 ppm in the extract with the concentration of 100 ppm.

### 3.3. LC/MS Evaluation

**Figure 4:**
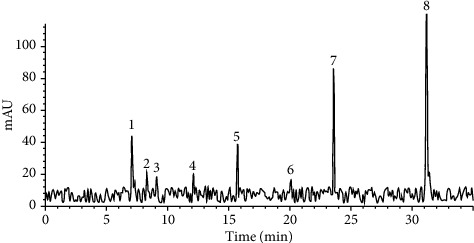
LC/MS analysis of the root of *Astragalus microcephalus*. Eight flavonoid glycosides were detected by LC/MS.

The *Astragalus* extract LC-ESI-MS analyses are shown in Figure 4. The identification of individuals' peak, retention time, concentration, and [*M* + *H*]^+^ are listed in Table 1. Based on the comparison of those values with system library data, 11 flavonoid peaks were identified, as shown in Figure 4 and Table 1. The maximum concentration was for formononetin and kaempferol-3-O-glucoside, which were isoflavone and flavonol, respectively.

### 3.4. Docking Result

Results from *in silico* study (Tables 2 and 3) revealed that kaempferol-3-O-glucoside has the highest effect on ERs *α* and *β* compared to other components. Then, kaempferol-3-O-sophoroside and formononetin have a high affinity to ERs *α* and *β*. The binding affinity of kaempferol-3-O-glucoside was −8.5 (Kcal/mol), and those of formononetin and kaempferol-3-O-sophoroside were −8 (Kcal/mol) to ER *β*. Kaempferol-3-O-neohesperidoside displayed binding energy of −7.9 to ER *α*, and the binding affinity of quercetin-3-O-neohesperidoside was −7.8 (Kcal/mol).

### 3.5. *In Vitro* Drug Release Study

**Figure 5:**
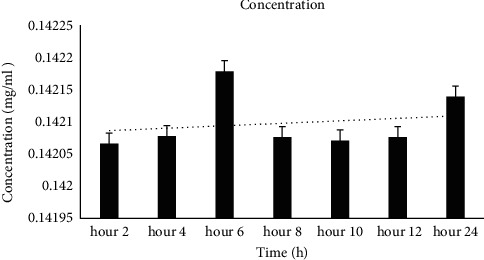
*In vitro* drug release study during 24 h at 37°C, at 415 nm. The absorbance was 0.1420 ± 0.0 mg/ml.

The quercetin absorbance at 415 nm was (*y* = 0.0166*x* + 0.143, *R*^2^ = 0.997), and by this formula, the flavonoid was passed through the membrane at a constant rate, and the amount was 0.142 mg/ml during the examination (24 h) (Figure 5).

### 3.6. MTT Assay

**Figure 6:**
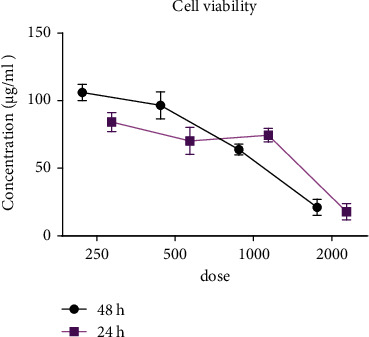
NHDF cell viability percent treated with different doses of 2,000, 100, 500, and 250 *μ*g of the extract (the 500 *μ*g/ml of the extract showed the maximum cell viability).

Cell viability results indicated extract toxicity at a dose of 2,000 *μ*g after 48 h (0.97% cell viability), and with doses of 250, 500, and 1,000 *μ*g, cell viabilities were over 100%, indicating cell growth. The maximum cell viability percent was at doses of 250 and 500 *μ*g, respectively, in 24 and 48 h (Figure 6). With these results, the 500 *μ*g dose was selected for the scratch wound healing study.

### 3.7. Evaluation of the *In Vitro* Wound Healing Activity

**Figure 7:**
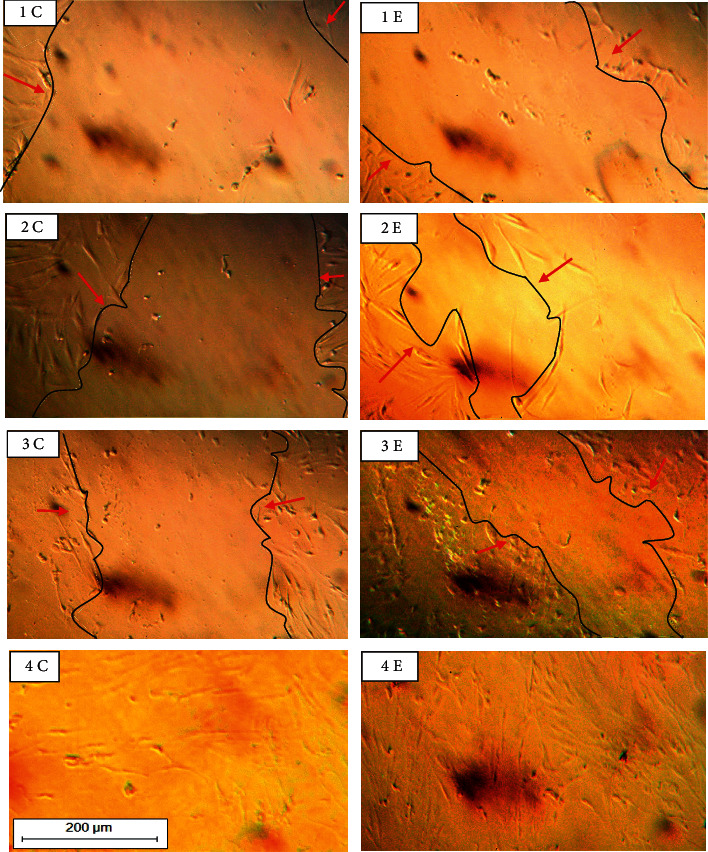
Microscopic images (100×) of scratch wound healing assay (measurement of the scratch closing area). Pictures 1, 2, 3, and 4, respectively, represent times 0, 2, 8, and 24 h after treatment. (E) is the extract group, and (C) is the control group. Red arrows and black lines represented the scratch margin.

The *in vitro* scratch closing analysis showed no mortality; moreover, the extract group showed an increase in the number of fibroblast cells compared to the control group. The extract group showed significantly better wound healing activity in each episode of time (*P* = 0.029). The extract group had closed the scratch better than the control group, especially in time 8 h after treatment (*P* = 0.029). During 24 h, the scratch was completely closed in both groups (Figure 7 and Table 4).

### 3.8. Wound Size Analysis

Wound evaluation showed that all groups had over 90% of wound healing after day 14. Initially, the wound healing ratio in the free groups was the highest, but over time, all groups speeded up in wound healing and showed better effects compared to the free group. To follow the pairwise comparisons of the groups, we used the Tukey test, the results of which showed that the average wound healing ratio on the 4th day for the free group was higher than that for the extract group (*P*=0.043). On day 7, the extract group was higher than the free (*P* ≤ 0.001) and silver (*P* ≤ 0.001) groups. The Vaseline group was more than the silver (*P*=0.004) and the free (*P*=0.008) groups. On the 10th day, the extract group was higher than the free group (*P*=0.007), and the Vaseline group was higher than the free group (*P*=0.003). On the 14th day, there was no statistically significant difference between the groups. Furthermore, after the treatment of the study group, the extract showed minimum wound scar (Tables 5 and 6).

### 3.9. Histopathological Evaluation

Histopathological results indicated that congestion, edema, inflammation, necrosis, and angiogenesis decreased during the examination, and fibroblast proliferation, fibrosis, and epithelization increased, especially in the extract group compared to the silver sulfadiazine and free groups. The Kruskal–Wallis test results showed a significant difference in epithelialization between groups (*P*=0.005). By performing a *post hoc* test, we found a significant difference between the extract and the free, silver (*P*=0.002), and Vaseline (*P*=0.04) groups; epithelialization was higher in the extract group.

The fibroblast proliferation was significant between groups, according to the results of the Kruskal–Wallis test (*P*=0.008). The *post hoc* test comparing the two groups showed that fibroblast proliferation between the silver and extract groups was significant (*P*=0.007) to the extent that this index was higher in the extract group. Fibroblast proliferation in the Vaseline group was higher than silver group (*P*=0.001). Kruskal-Wallis test in groups was also significant for fibrosis (*P*=0.002). Fibrosis in the free group was higher than that in the silver group (*P*=0.014), and the extract group was higher than the silver group (*P* < 0.001). Moreover, the amount of fibrosis in the extract group was higher than that in the Vaseline group (*P*=0.014). Angiogenesis was significant between treatment groups (*P*=0.004). Angiogenesis in the extract group was higher than that in the Vaseline group (*P*=0.013), and the extract group was higher than the silver group (*P* < 0.001). Furthermore, the free group was higher than the silver group (*P*=0.037).

After a significant Kruskal–Wallis congestion test between groups (*P*=0.002) with a *post hoc* test, it was found that congestion in the extract group was less than that in the free (*P*=0.012) and silver (*P*=0.005) groups, and the Vaseline group was less than free group (*P*=0.012) and silver (*P*=0.005). The Kruskal–Wallis test result for edema showed a significant difference between the groups (*P*=0.034). This index was lower in the extract group than the free (*P*=0.009), Vaseline (*P*=0.029), and silver groups (*P*=0.021). Inflammation was also significant between the groups (*P*=0.009), so this index was lower in the extract group than in the Vaseline (*P*=0.008), silver (*P*=0.002), and free (*P*=0.031) groups. Necrosis was also significant among groups (*P*=0.013). By examining the two comparisons, we found that the amount of necrosis in the extract groups was less than that in the Vaseline (*P*=0.01) and silver (*P*=0.002) groups.

In Masson's trichrome dye samples, the blue zone showed proliferation of tissue, which can be attributed to collagen bond formation. On day 14, The Vaseline and extract groups showed a higher blue zone (Table 7).

## 4. Discussion

A wound is a high-prevalence complication that affects society. Its treatment becomes more important when associated with factors such as the length and depth of the wound, wound infections, and other underlying diseases such as diabetes [5].

Generally, factors affecting wound healing are classified into local and systematic factors. Local factors directly affect the wound, and systematic ones affect the general health or condition of individuals and their ability to recover. Furthermore, many other factors are related to both local and systematic factors [29, 30]. Infection is an important local factor, and microorganisms normally exist on the surface of the skin. When the skin is wounded, they have access to the underlying tissues. Factors such as state of infection, replication status, and microorganism loading in the tissue represent the classification of wound infection, such as microbial contamination, colonization, local infection, and systematic separation of invasive infection [6, 31].

Inflammation is a normal body response in the wound healing process, and microbial contamination prolongs this response. Both bacteria and endotoxins elevate proinflammatory cytokines such as TNF-*α* and interleukin-1. Furthermore, inflammation increases protease enzyme in the tissue, which degrades growth factors rapidly. If this situation continues in the wound, it leads to failure in healing*. Staphylococcus aureus* (*S*. *aureus*) and *Pseudomonas aeruginosa* (*P*. *aeruginosa*) are important causes of wound infection [32, 33]. ERs are distributed in various organs in the body. The skin is an important target for estrogen by having two ERs (*α* and *β*). Research has shown that estrogens increase mitotic activity in the epidermis and can be used in wound complications [34]. Studies have reported that the rate of wound healing in females has been much higher than in males. Furthermore, the rate of wound healing has decreased in elderly individuals; this observation is directly related to estrogen hormones [35].

Flavonoids and glucosides are the second important metabolites usually present in most plants. They are especially found in the roots of the Leguminous family. A subclassification of flavonoids is isoflavonoids. They have a similar structure to estrogen and can bind to estrogen-related receptors. *Astragalus* species belong to the Legumes family and are reported to have flavonoids [30]. In the current study, herbal analysis of the extract showed a high amount of flavonoid content, and LC/MS evaluation represented some flavonoids and glycoside isoflavonoids with the base structure of rutin, quercetin, kaempferol, and calycosin. Although the presentation of genistein in the literature was high and could be isolated from this genus [36], in this study, the genistein presentation of the extract was traced; it was 1.2449 ppm in the extract.

ER are the target for the body's estrogens. These receptors can be used for designing molecules with a high binding affinity to ERs to utilize them for estrogen-related diseases. The function varies from agonist and antagonist mechanisms in the body based on the designed structure.

Various compounds can affect ERs *α* and *β* (estrogens, some androgens, phytoestrogens, antiestrogens, and environmental estrogens) because of intrinsic ERs *α* and *β* ligand-binding domain (LBD) plasticity [37].

ER *α* LBD structure contains 11 *α*-helices. The attachment to this enzyme differs from the hydrogen bond for the steroid/hormone ligand and hydrophobic interactions for the nonsteroidal ligand. Estradiol hydroxyl groups as in positions 3 and 17 of the *A* and *D* rings have hydrogen bonded to Glu353 from H3, Arg394 from H5, and a water molecule and His524 from H11. ER *β* can bond to genistein and estradiol with hydrogen bonds of hydroxyl moieties with histidine groups of the receptor.

Studies have shown that if a special substance tends to affect ERs *α* and *β* as an agonist or relative agonist, it should be placed at the interaction distance of the active site of amino acids Glu353, Arg394, and His524. However, it differs for an antagonist, and just one missing interaction can have an opposite effect [38]. To estimate the physiological behavior of ligands in the vicinity of enzymes, the binding affinity of ligands to receptors is important. In this study, estradiol (a body estrogen) as an agonist and finasteride as an antagonist of ERs were evaluated to find the binding affinity to ERs *α* and *β* as standard compounds and for further investigation with LC/MS suggested compounds. Furthermore, in the current study, we observed *H* bonds between active site amino acids from ER *α* Glu353, Arg394, and His524. Moreover, for ER *β*, the bond and conformation with the lowest energy (highest binding affinity) were selected. For ER *α*, all compounds have conformations near the interaction distance of the active site. Furthermore, due to the flexible structure of ERs, the compounds showed H bonds to ALA 497 of RE *β*.

The binding affinity for the estradiol to the ERs *α* and *β* is −6.822 ± 0.03 and −6.544 ± 0.13 (Kcal/mol), respectively. The value for finasteride is −6.956 ± 0.06 and −6.578 ± 0.12 (Kcal/mol). Kaempferol-3-O-glucoside has the highest effect on ERs *α* and *β* compared to other components, given that this affinity is higher than that of finasteride and estradiol.

Kaempferol-3-O-glucoside, kaempferol-3-O-sophoroside, kaempferol-3-O-neohesperidoside, and quercetin-3-O-neohesperidoside have significantly higher binding affinities (lower energy bond) to ER *α* compared to estradiol and finasteride. Moreover, these compounds, including calycosin-7-O-beta-D-glucoside, have significantly higher binding affinities to ER *β* compared to estradiol and finasteride (Table 2). The binding affinity details of compounds can be obtained in Table 2. In the Pang et al. study, genistein, daidzein, and kaempferol exhibited different levels of antagonistic activity against ER *α* [39]. In other studies, it was found that kaempferol functions as an estrogen competitor and the binding affinity for this compound is −7, which is−6.7 (Kcal/mol) for estrogen. This finding is similar to the current study [40]. Ganguly et al. evaluated some polyphenols and flavonoids on estrogens receptors. They found that catechin and epicatechin showed the best binding affinity toward ER *α* and suggested that they may affect the reproductive hormone homeostasis [41]. Ye et al. found that the proliferation of Caco-2 cells (ER*β* expressing cells) increased when treated with isoflavone, and it would be much more by hydroxylated isoflavone biotransformation products. This could mediate the progression of ER*β*-expressing gut (e.g., colon) cancers.

Despite the contradictory effects of oral use of estrogen-like compounds (flavonoids, especially isoflavonoids), the present study showed that these compounds significantly affect wound healing in both *in vivo* and *in vitro* conditions. The mechanism of this wound healing effect is due to the presence of ERs *α* and *β* in the target tissue (skin) and the specific structure of this group of compounds. Furthermore, the *in silico* study confirmed that the high affinity of these compounds to ERs could be a possible mechanism for this effect. This finding was consistent with Özay et al.'s study, which showed that kaempferol can heal wounds in the diabetic and nondiabetic models [42].

Other studies evaluated the effect of *Astragalus membranaceus* (Fisch.) Bunge wound healing effect *in vitro* and *in vivo* and in both conditions, showing wound healing activity [43, 44]. Rat, mice, and Guinea pigs were usually used for wound healing assay in the *in vivo* condition. The trial duration was normally 7, 14, and 21 days according to observations and each study's considered parameters [45, 46]. Silver sulfadiazine and phenytoin ointment were usually used as standard medicine groups [47–49]. Some other studies used mupirocin and povidone-iodine ointment [50, 51]. In this study, a 21-day trial was conducted to evaluate wound healing in the *in vivo* and silver sulfadiazine condition selected as standard medicine.

By examination of drug release from full-thickness skin, the rate of drug delivery to the tissue can be calculated. By measuring the amount of flavonoid crossing the skin, the release rate of the drug was observed and showed zero kinetics of extract absorbance, causing the Vaseline-based formula to cross the skin with a constant amount. Furthermore, as Vaseline was not removed easily from the dorsal skin of rats, the daily treatment with this formula was better than drug delivery during daily application. Thereby, the duration of contact of the extract with the wound is longer, and the healing process is completed better.

In the present study, topical application of *Astragalus* significantly enhanced the wound healing rate as assessed by the increase in collagen synthesis in wound tissues, a finding similar to Qian et al.'s study [52]. Histological findings also showed enhanced proliferation of fibroblasts and epithelialization; they were significantly better in the extract group than in other groups. Hydroxyproline level measurement of the wound is a biomarker of the amount of collagen in the tissues (cellular repair building materials) [53]. Moreover, in the current study, collagen was evaluated by Masson's trichrome stain, and fibrosis indicated collagen bonds.

Afonso et al. evaluated the *in vitro* wound healing activity by scratch assay on the NHDF cell line [54]. The rate of closing the wound was measured, and cells' migration demonstrated the wound healing. In the current study, the medium dose of treatment was selected based on toxicity evaluation by MTT assay. Obviously, wound migration and closing of the scratch were observed, especially in the extract group, for 8 h after treatment with the *Astragalus* extract in comparison with control samples, which indicated a faster rate of wound healing.

Overall, for future studies, it is suggested to measure the proinflammatory cytokine to evaluate the wound inflammation and study the extract's antimicrobial and antifungal activity. Due to the limitation of our study to isolate isoflavone glycoside from the extract, it is better to evaluate each of the LC/MS result compounds for wound healing analysis in future studies.

## 5. Conclusion

*Astragalus microcephalus* showed wound healing activity *in vivo* and *in vitro* due to flavonoids (especially isoflavonoids) presented in the extract. These flavonoids have a high affinity for ERs *α* and *β*, act similar to estrogen in the skin tissue, and accelerate the wound healing process.

## Figures and Tables

**Table 1:** The LC/MS peaks information, retention time (RT) values, [*M* + *H*] ^+^, and concentration.

Peak IDs	RT (min)	Recovery (%)	*m*/*z* [*M* + *H*]+	Concentration (*μ*g/g)
1. Calycosin-7-O-beta-D-glucoside	7.08	95	447	65.49085
2. Quercetin-3-O-sophoroside	8.31	97	626	31.6446
3. Quercetin-3-O-neohesperidoside	9.1	99	609	27.42942
4. Kaempferol-3-O-sophoroside	12.1	96	770	29.71406
5. Kaempferol-3-O-neohesperidoside	15.72	99	594	57.51008
6. Kaempferol-3-O- neohesperidoside-7-O-rhamnoside	20.1	99	595	24.84754
7. Kaempferol-3-O-glucoside	23.6	101	593	128.8624
8. Formononetin	31.19	100	269	207.7947

Calycosin-7-O-beta-D-glucoside, quercetin-3-O-sophoroside, quercetin-3-O-neohesperidoside, kaempferol-3-O-sophoroside, kaempferol-3-O-neohesperidoside, kaempferol-3-O- neohesperidoside-7-O-rhamnoside, kaempferol-3-O-glucoside, and formononetin came out of the LC column, respectively.

**Table 2:** Flavonoids present in the extract and their affinity to binding to the ERs *α* and *β*.

Ligand	ER *α* (Kcal/mol)	ER *β* (Kcal/mol)
Estradiol	−7.7 to −6.7	−7.5 to −6.4
Finasteride	−7 to −6.8	−8.2 to −6.6
Formononetin	−7.2 to−6.4	−8 to −6.2
Kaempferol-3-O-glucoside	−7.9 to −6.9	−8.5 to −7.1
Kaempferol-3-O-sophoroside	−7.4 to −6.7	−8.0 to −7.4
Kaempferol-3-O-neohesperidoside	−7.9 to −7.3	−7.3 to −6.8
Quercetin-3-O-neohesperidoside	−7.8 to −6.8	−7.9 to−7.3
Calycosin-7-O-beta-D-glucoside	−7.6 to −7	−7.7 to −7.2

Molecular docking results indicated that all ligands had a high affinity (<−6.3) to receptors.

**Table 3:** Interaction between amino acids in the binding site of ERs *α* and *β*, and standard agonist (estradiol), standard antagonist (finasteride), and components (calycosin-7-O-beta-D-glucoside, formononetin, kaempferol-3-O-neohesperidoside, and kaempferol-3-O-sophoroside) identified by LC/MS.

Compound name	ER *α*	ER *β*
Estradiol	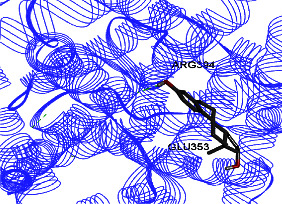	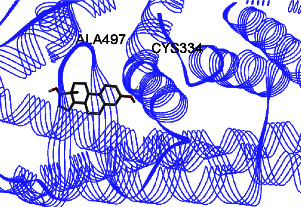
Finasteride	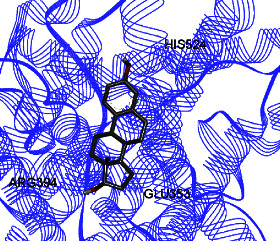	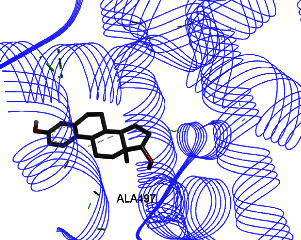
Calycosin-7-O-beta-D-glucoside	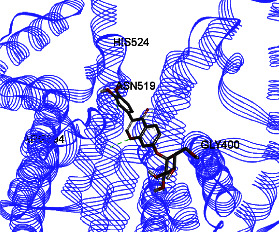	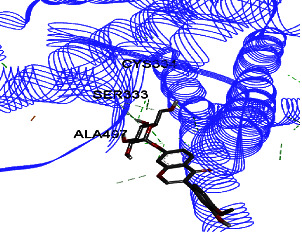
Formononetin	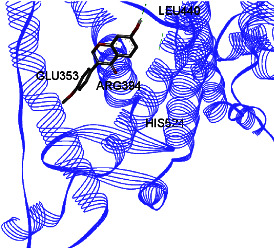	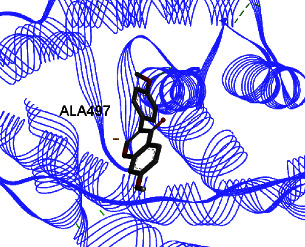
Kaempferol-3-O-neohesperidoside	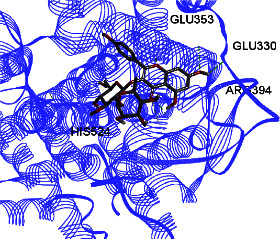	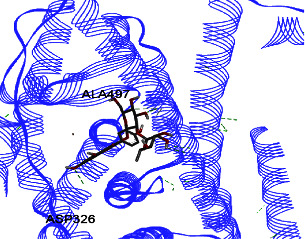
Kaempferol-3-O-sophoroside	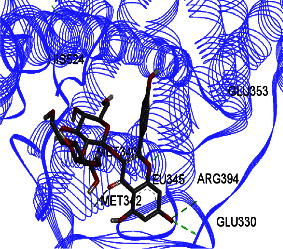	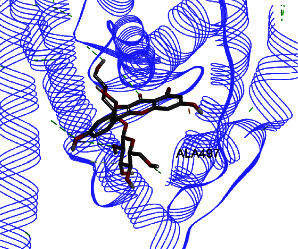

The green bonds show intramolecular hydrogen bonds.

**Table 4:** Calculation of scratch closing area and percentage of growth at 0, 2, 8, and 24 h after treatment.

Time (h)	Area		Percent of growth	
	Extract	Blank	Extract	Blank
0	630.5	857.24	7.88%	18.37%
2	584.4	699.75		
			89.43%	23.23%
8	61.75	537.18		
			53.457%	45.76%
24	28.74	291.34		

Percent of growth = −(*T*2 scratch area − *T*1 scratch area)/*T*1 scratch area × 100.

**Table 5:** Wound healing ratio (the ratio of wound healing = (area of original wound − area of remaining wound)/area of original wound × 100), in the silver sulfadiazine, free, Vaseline, and extract groups during the study on days 4, 7, 10, and 14.

	Extract	Vaseline	Silver	Free	*P*
Day 4	32.78 ± 2.63	41.88 ± 3.27	34.91 ± 5.44	42.40 ± 5.68	0.020
Day 7	73.81 ± 3.13	66.65 ± 1.62	54.04 ± 4.52	55.05 ± 5.81	<0.001
Day 10	85.90 ± 4.08	87.82 ± 5.92	81.75 ± 2.99	72.74 ± 4.84	0.003
Day 14	93.32 ± 3.36	91.29 ± 7.66	89.74 ± 1.33	85.61 ± 5.45	0.224

**Table 6:** Regarding wound analysis, groups were treated with extract, Vaseline, and silver sulfadiazine, and the free group did not receive anything on days 1, 4, 7, 11, 14, and 21.

Day	Extract	Vaseline	Silver sulfadiazine	Free
1	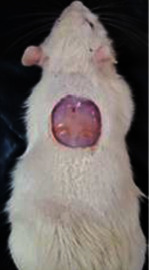	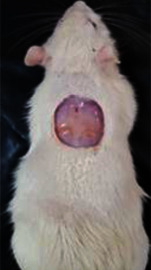	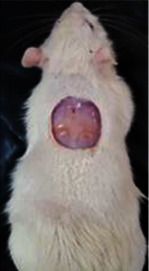	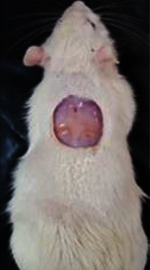
4	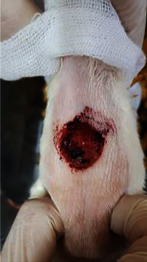	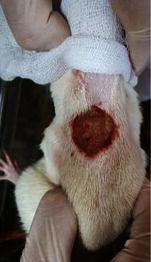	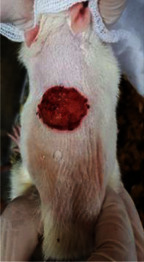	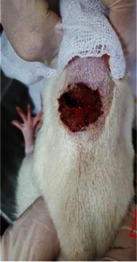
7	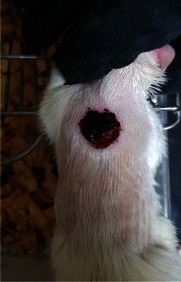	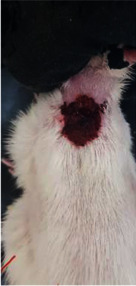	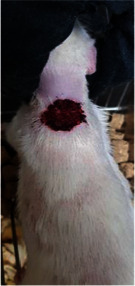	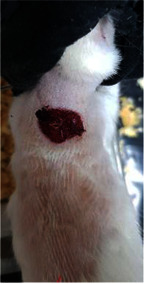
11	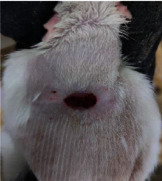	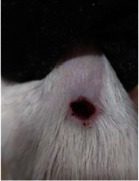	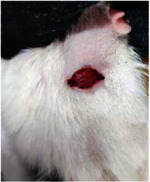	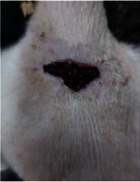
14	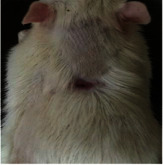	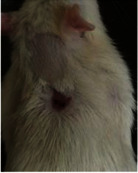	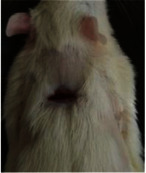	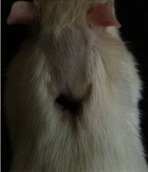
21	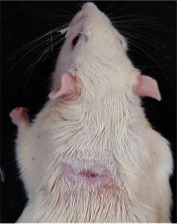	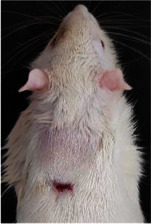	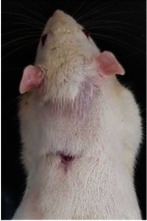	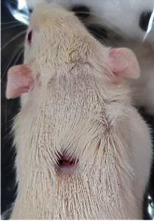

On day 1, the full-thickness wound was induced in all groups, and there was no difference between groups.

**Table 7:** Microscopic images (40×) of different treatment groups' tissue samples after being stained with hematoxylin and eosin (*H* & *E*) and Masson's trichrome (MT) on days 4, 7, 14, and 21 after treatment (blue zone indicates the fibroblast proliferation).

Groups	Day 4	Day 7	Day 14	Day 21
Extract (*H* & *E*)	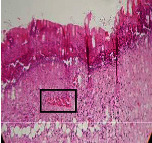	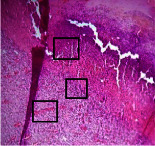	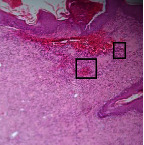	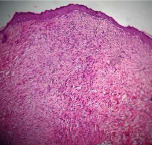
Extract (MT)	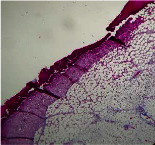	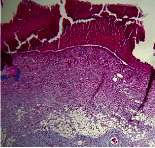	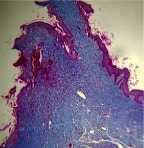	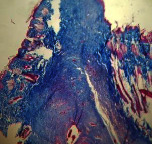
Vaseline (*H* & *M*)	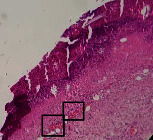	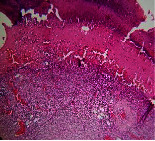	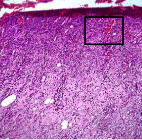	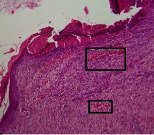
Vaseline (MT)	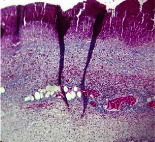	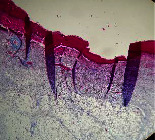	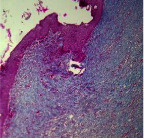	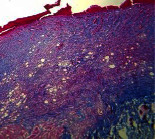
Silver (*H* & *E*)	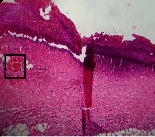	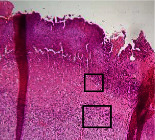	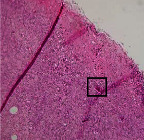	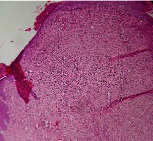
Silver (MT)	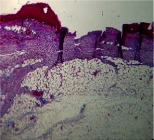	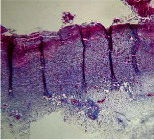	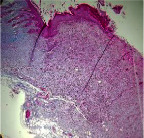	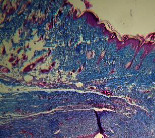
Free (*H* & *E*)	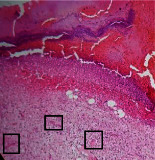	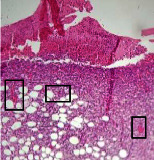	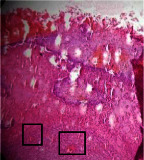	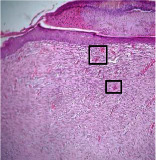
Free (MT)	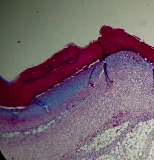	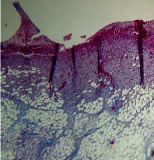	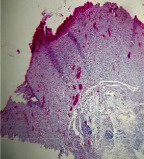	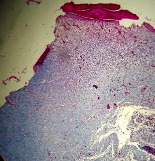

Squares represent morphological changes such as congestion, edema, inflammation, necrosis, and angiogenesis.

## Data Availability

All materials, such as rats, silver sulfadiazine, Vaseline, microbial medium cultures, and ethanol for extraction, were provided by the Mazandaran University of Medical Science. Data were obtained from laboratories of the Mazandaran University of Medical Science, such as pathology and serology laboratories.
